# Supplementary figures and images for: A novel SUN1-ALLAN complex coordinates segregation of the bipartite MTOC across the nuclear envelope during rapid closed mitosis in Plasmodium berghei
Source: eLife. 2025 May 20;14:RP106537. doi: 10.7554/eLife.106537 (PMC12092005; doi:10.7554/eLife.106537)

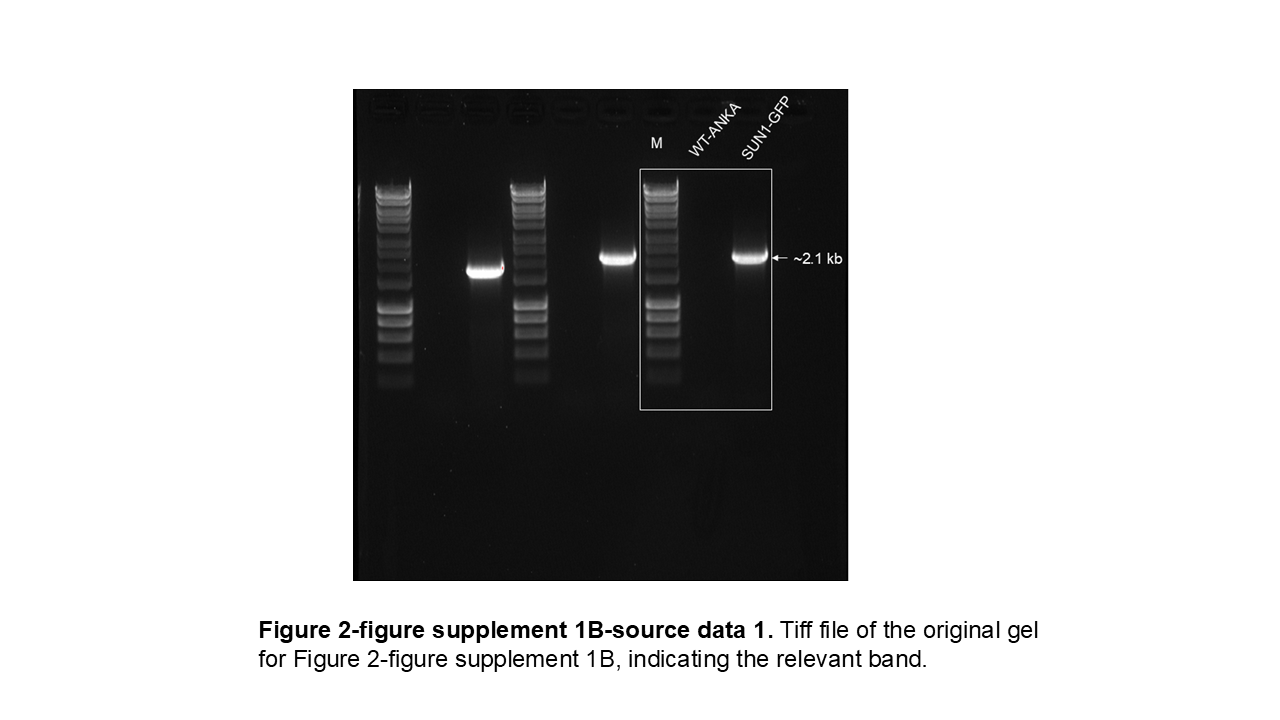

Supplement: Figure 2—figure supplement 1—source data 1. [file elife-106537-fig2-figsupp1-data1.zip › Figure 2-figure supplement 1 -source data-1-labelled/Sun1-GFP-gel-labelled (2).TIF]

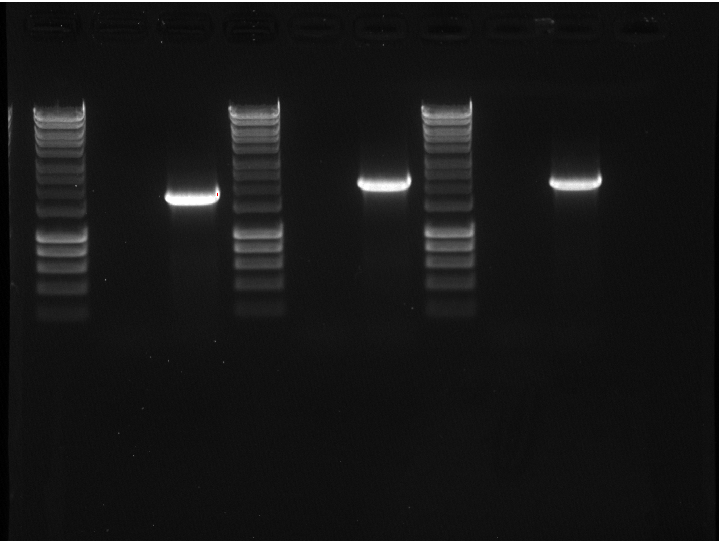

Supplement: Figure 2—figure supplement 1—source data 2. [file elife-106537-fig2-figsupp1-data2.zip › Figure 2-figure supplement 1-source data1/Sun1-GFP-gel.tif]

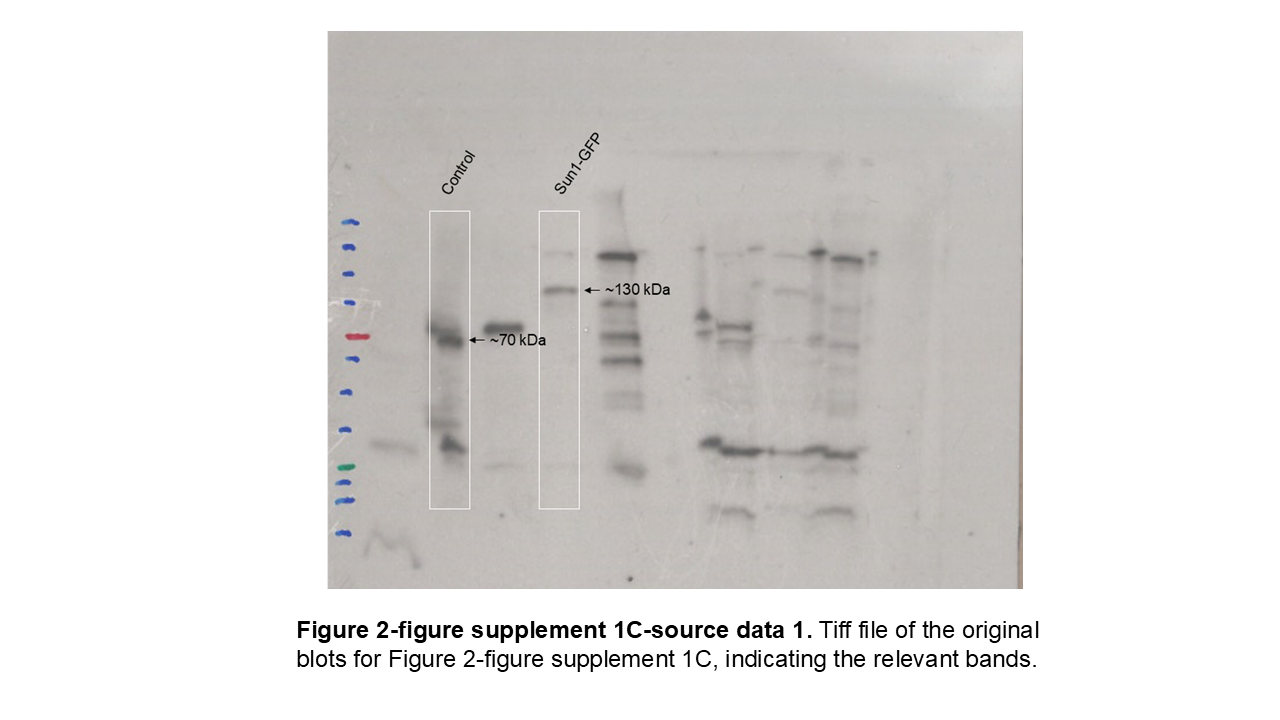

Supplement: Figure 2—figure supplement 1—source data 3. [file elife-106537-fig2-figsupp1-data3.zip › Figure 2-figure supplement 1 source data-2- labelled/Sun1-GFP-WB-labelled.TIF]

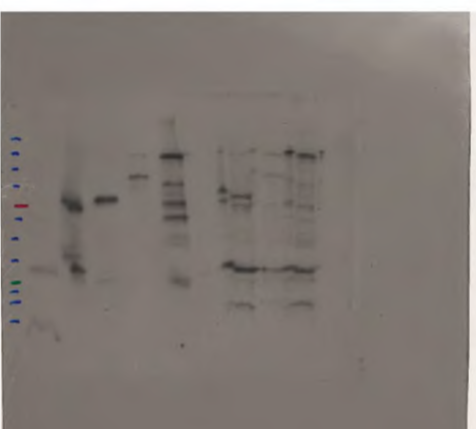

Supplement: Figure 2—figure supplement 1—source data 4. [file elife-106537-fig2-figsupp1-data4.zip › Figure 2-figure supplement 1-source data-2/Sun1-GFP-WB.tif]

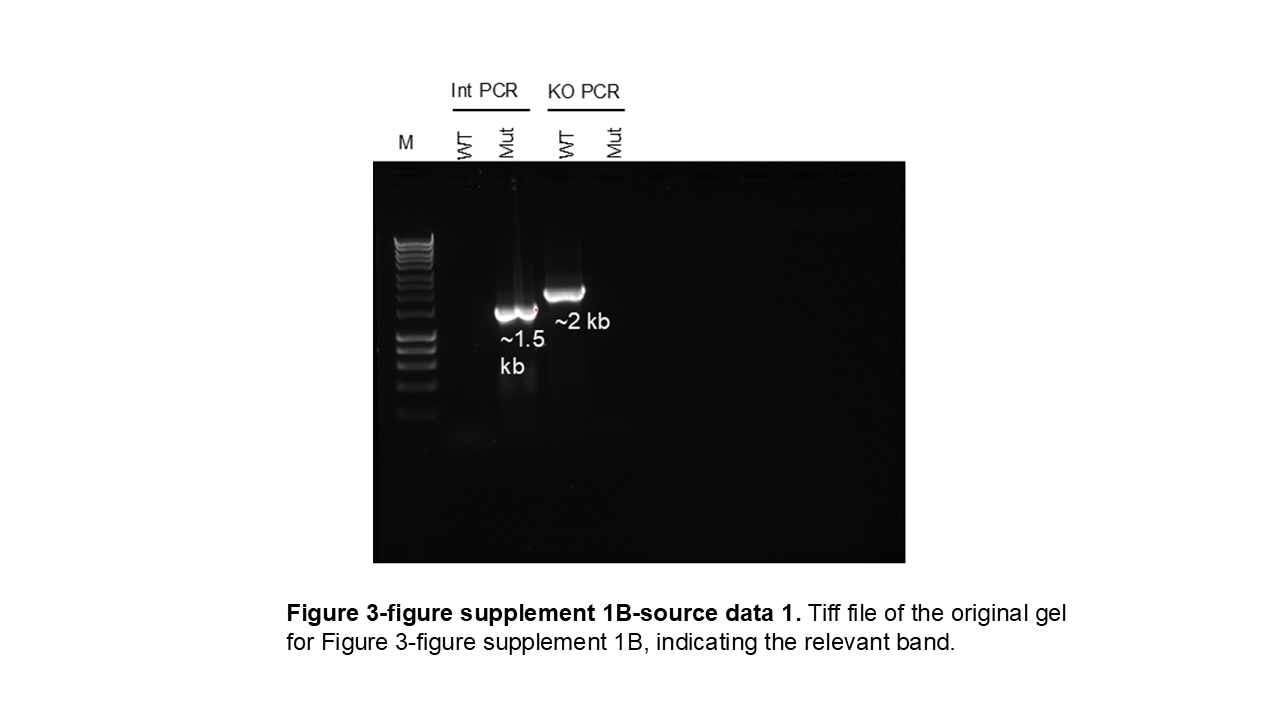

Supplement: Figure 3—figure supplement 1—source data 1. [file elife-106537-fig3-figsupp1-data1.zip › Figure 3-figure supplement 1 -source data-1- labelled/Sun1-KO-gel-labelled (2).TIF]

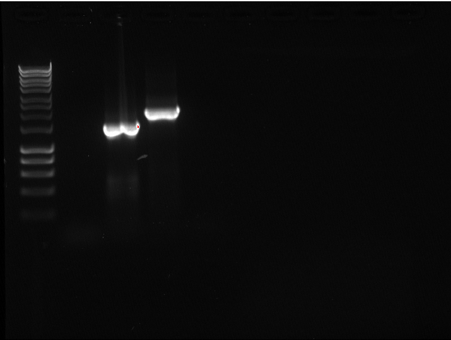

Supplement: Figure 3—figure supplement 1—source data 2. [file elife-106537-fig3-figsupp1-data2.zip › Figure 3-figure supplement 1-source data-1/Sun1-KO-gel.tif]

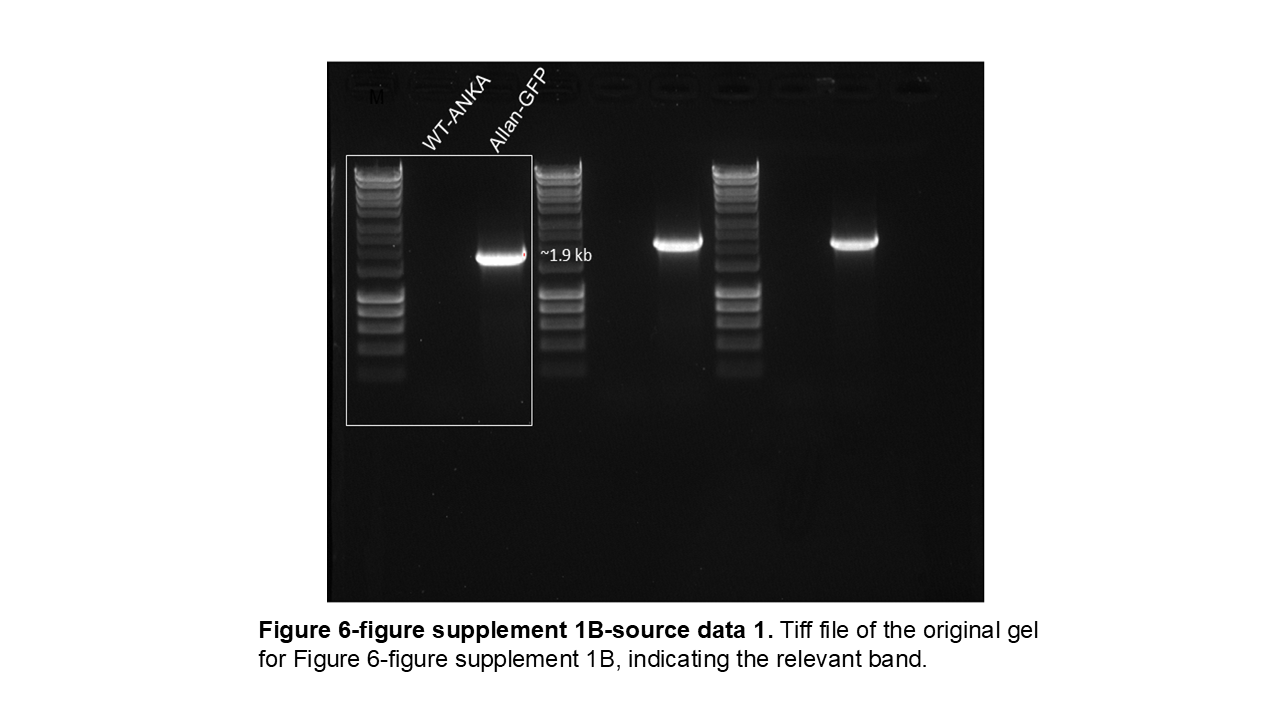

Supplement: Figure 6—figure supplement 1—source data 1. [file elife-106537-fig6-figsupp1-data1.zip › Figure 6-figure supplement 1 -source data-1- labelled/Allan-GFP-gel-labeleld.TIF]

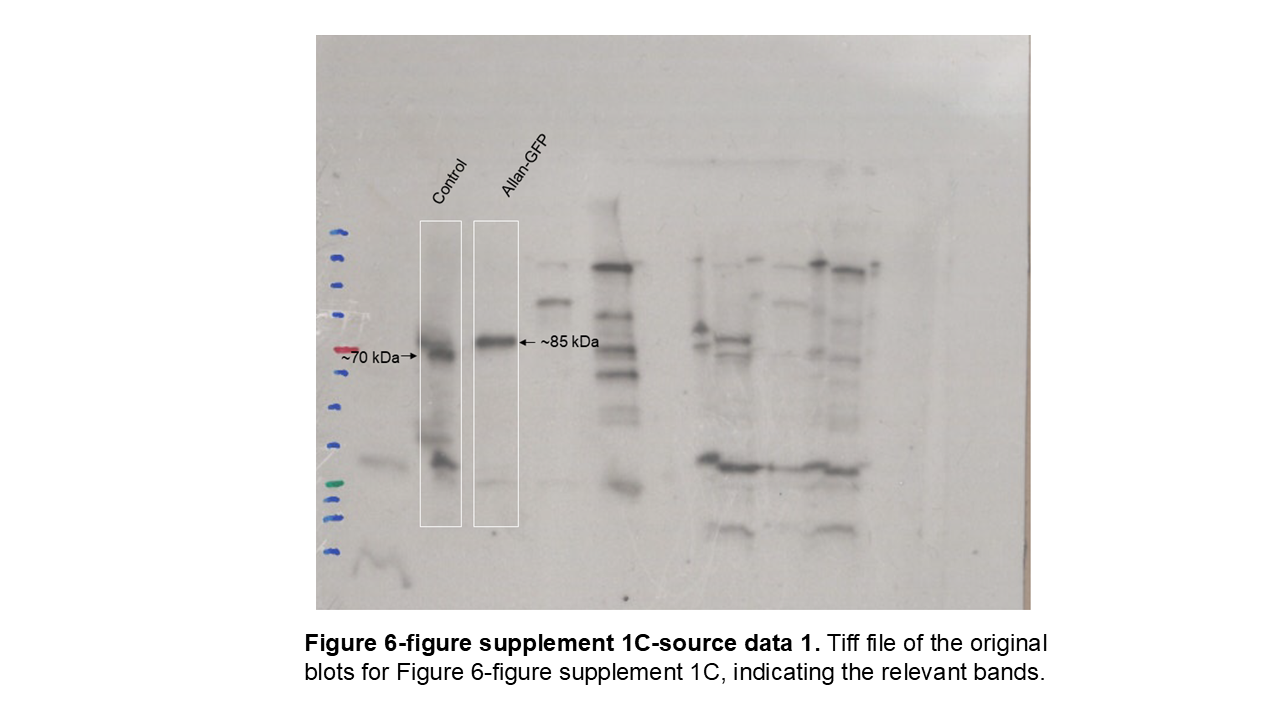

Supplement: Figure 6—figure supplement 1—source data 3. [file elife-106537-fig6-figsupp1-data3.zip › Figure 6-figure supplement 1 -source data-2- labelled/Allan-GFP-WB-labelled (2).TIF]

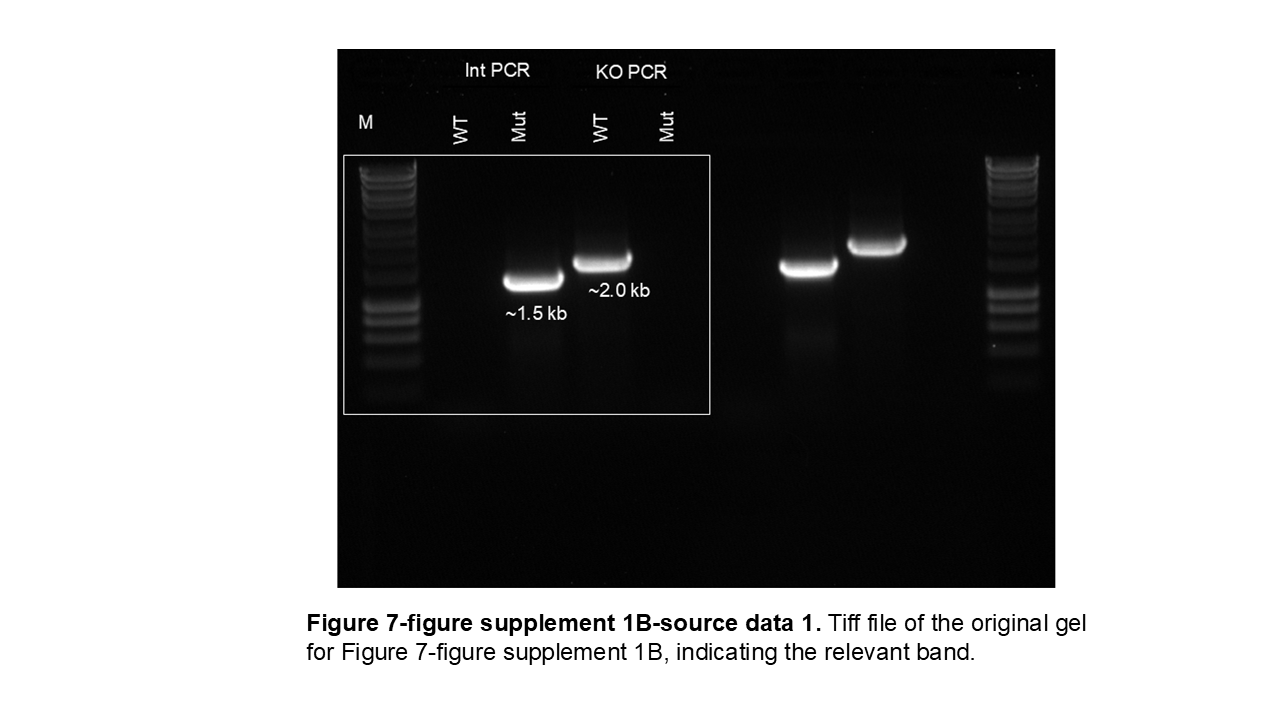

Supplement: Figure 7—figure supplement 1—source data 1. [file elife-106537-fig7-figsupp1-data1.zip › Figure 7-figure supplement 1 - source data-1-labelled/Allan-KO-gel-labelled (2).TIF]

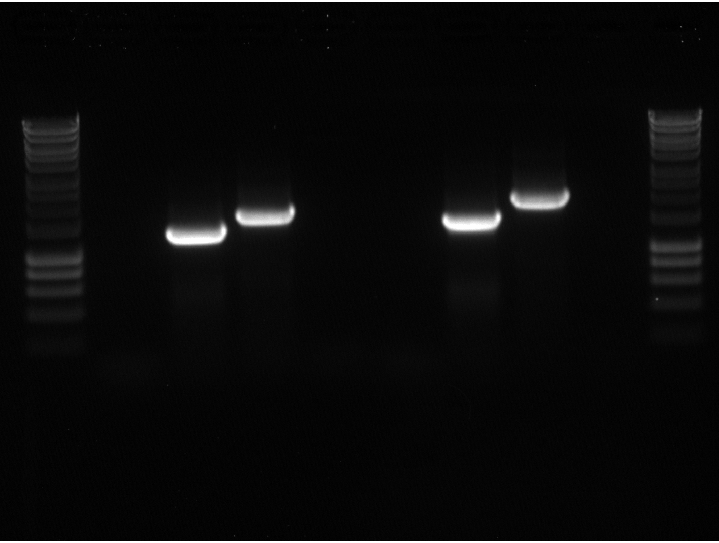

Supplement: Figure 7—figure supplement 1—source data 2. [file elife-106537-fig7-figsupp1-data2.zip › Figure 7-figure supplement 1-source data-1/Allan-KO-gel.tif]
